# Achieving complete metabolic response in stage IV lung adenocarcinoma with chemotherapy, nivolumab, ipilimumab, and salvage SBRT: A case report

**DOI:** 10.1002/rcr2.1362

**Published:** 2024-04-24

**Authors:** Salvador Gamez Casado, Javier David Benitez Fuentes, Beatriz Álvarez Rodríguez, Gema García Ledo

**Affiliations:** ^1^ Hospital Universitario HM Sanchinarro, Centro Integral Oncológico Clara Campal (HM‐CIOCC) Department of Medical Oncology Madrid Spain; ^2^ Hospital General Universitario de Elche Department of Medical Oncology Elche Spain; ^3^ Hospital Universitario HM Sanchinarro, Centro Integral Oncológico Clara Campal (HM‐CIOCC) Department of Radiation Oncology Madrid Spain

**Keywords:** adenocarcinoma, dual checkpoint inhibition, immunotherapy, lung cancer, metastatic

## Abstract

Oncogene‐negative, PDL1‐negative metastatic non‐small cell lung cancer (NSCLC) presents significant treatment challenges due to its complexity and resistance to conventional therapies. The case report presented addresses a 55‐year‐old male patient with oncogene‐negative, PDL1‐negative stage IV lung adenocarcinoma, showcasing an exceptional complete metabolic response to a multimodality treatment combining double immune checkpoint inhibition (ICI) and chemotherapy, followed by salvage stereotactic body radiotherapy (SBRT). The patient underwent a treatment regimen incorporating two cycles of carboplatin, pemetrexed, nivolumab, and ipilimumab followed by nivolumab, and ipilimumab maintenance. After a partial response, SBRT was applied to persistent lesions, achieving a complete metabolic response. This case highlights the potential of combining dual ICI with chemotherapy and SBRT in treating oncogene‐negative, PDL1‐negative NSCLC underscoring the importance of multimodality treatment strategies.

## Graphical abstract

A 55‐year‐old male with stage IV oncogene‐negative, PDL1‐negative lung adenocarcinoma demonstrated a complete metabolic response to a novel treatment strategy. This regimen combined double immune checkpoint inhibition (ICI) with chemotherapy, followed by stereotactic body radiotherapy (SBRT), after initial partial response to two cycles of carboplatin, pemetrexed, nivolumab, and ipilimumab, plus maintenance with nivolumab and ipilimumab. This case underscores the effectiveness of integrating multiple treatment modalities for non‐small cell lung cancer (NSCLC).

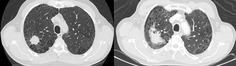

## INTRODUCTION

Non‐small cell lung cancer (NSCLC), frequently diagnosed at advanced stages, poses considerable treatment challenges attributed to its inherent complexity and resistance to conventional therapies.^1^ A significant proportion (over half of the patients with NSCLC) is identified at advanced stages, underscoring the urgency for more effective treatment strategies.^1^ While the advent of targeted therapy and immunotherapy has revolutionized the management of NSCLC, leading to notable improvements in survival rates, patients with stage IV oncogene‐negative, PDL1‐negative NSCLC still face a poor prognosis.^1^ The present case report delves into such a scenario, demonstrating a durable complete metabolic response in a patient with advanced NSCLC combining double immune checkpoint inhibition (ICI) with chemotherapy together with SBRT for residual lesions The case underscores the emerging potential of combined immune checkpoint inhibition with radiotherapy, an approach that can lead to favorable outcomes in challenging cases of stage IV lung adenocarcinoma.

## CASE REPORT

**FIGURE 1:**
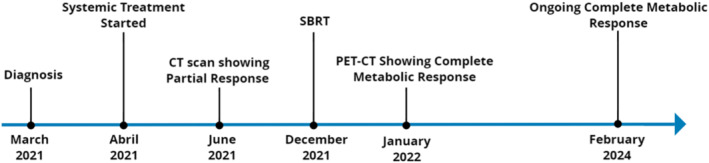
Timeline of events.

A 55‐year‐old Caucasian ex‐smoker male with an extensive history of previous smoking, quantified at an 80‐pack‐year, with no other medical background, presented with respiratory symptoms leading to diagnostic investigations in March 2021. The clinical course, response assessment, and follow‐up is summarized in a detailed timeline (Figure 1).

Following imaging and biopsy, he was diagnosed with TNM 8th edition T4N2M1c stage IV lung adenocarcinoma. A PET‐CT scan was conducted at an external radiology center, from which we obtained only a written report and were unable to access the images directly. The PET‐CT report described various contrast avid lesions, a 3 cm espiculated nodule in the right upper lobe (RUL), subcentimetric nodules in the right upper lobe, the right lower lobe, and the left lower lobe, a 24 mm right paratracheal adenopathy, a 16 mm right hilar adenopathy and two splenic lesions. The tumour sample tested negative for ALK, ROS1, and EGFR mutations, making him ineligible for targeted therapies, and exhibited PDL1 lower than 1%. A genetic analysis using OncoSELECT ctDNA liquid biopsy was performed showing no alterations in NRAS, DDR2, KRAS, AKT1, MAP2K1, ERBB2, ALK, MET, BRAF, FGFR1, ROS1, RET, NTRK, NTRK3, and EGFR.

Considering the advanced stage and molecular profile of the tumour, in April 2021 the patient started a treatment regimen comprising carboplatin AUC 5 and pemetrexed 175 mg/sqm every 3 weeks with folic acid and vitamin B12 supplementation for two cycles, along with nivolumab 360 mg every 3 weeks, and ipilimumab 1 mg/kg every 6 weeks until disease progression or unacceptable toxicity following the regimen of the CheckMate 9LA trial.^2^

**FIGURE 2:**
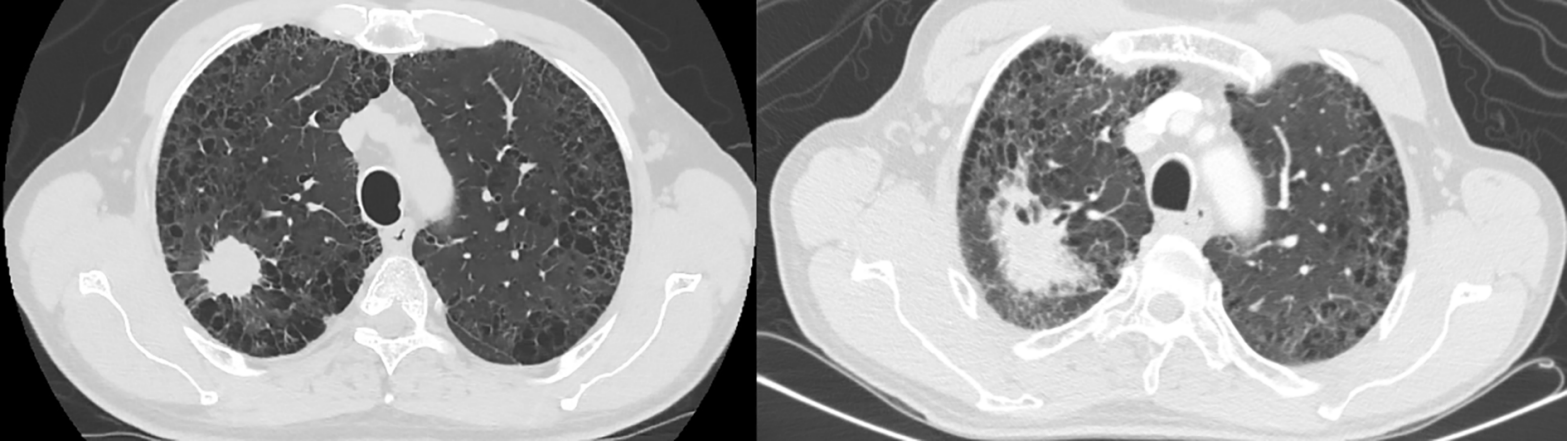
Pre‐treatment CT image from March 2021 (left) and the last CT image from November 2023 (right).

The patient achieved a complete radiological response of all the lesions except the RUL nodule and the right hilar adenopathy in the first CT scan performed in June 2021. After evaluating the case in a multidisciplinary committee, together with radiation oncologists, we decided to perform radical treatment with stereotactic body radiotherapy (SBRT) to the RUL lesion and the right hilar adenopathy. The SBRT was performed in December 2021, delivering 60 Gy in 5 fractions and 50 Gy in 10 fractions, respectively. Our radiotherapy doses were tailored for high‐precision SBRT, aiming for radical treatment while trying to ensure organ preservation, consistent with our center's protocols. We achieved a complete metabolic response after that, as was shown in the report of a PET‐CT scan performed in the same external radiology center. The pre‐treatment CT image from March 2021 and the last CT image from November 2023 are presented in Figure 2, showcasing the response to the treatment. The residual lesion with surrounding radiation pneumonitis has remained unchanged since after SBRT treatment was given.

The patient's treatment adherence was noteworthy. Toxicities experienced were minimal. They included grade 1 pruritus, and grade 1 amilase elevation. After 2 years of maintenance therapy with nivolumab and ipilimumab, we decided to continue the treatment. Our decision to continue the therapy was influenced by the personal experience and preference of the patient. Despite the acknowledged scarcity of evidence for extended treatment, the patient chose to remain on the same treatment, highlighting a lack of clinically significant toxicities and a sustained quality of life. The feasibility of this choice was supported by the coverage of treatment costs by his health insurance. As of the time of this report, the patient has remained on maintenance therapy for 3 years with no evidence of disease progression.

## DISCUSSION

The treatment of NSCLC, particularly in patients with oncogene‐negative and PDL1‐negative NSCLC, presents a significant clinical challenge.^1^ The exceptional durable metabolic complete response observed in this case of stage IV lung adenocarcinoma treated with dual ICI, chemotherapy, and SBRT, provides significant insights into the evolving landscape of lung cancer treatment.

The role of PD‐L1 expression in predicting the efficacy of combined PD‐1 and CTLA‐4 blockade therapy is complex and multifaceted. Initial studies suggested improved outcomes with this combination in tumours expressing PD‐L1, but clinical trials have shown varied results demonstrating modest improvements in response rates and survival over nivolumab alone in PD‐L1 ≥ 1% tumours while other studies did not find significant benefits.^3^ Intriguingly, the absence of PD‐L1 expression could be a key marker for the effectiveness of the combination PD‐1 and CTLA‐4 blockade.^2^, ^3^ This suggests that combination therapy could be more advantageous for patients with no PD‐L1 expression in settings similar to our case report.^3^

The integration of SBRT for persistent lesions underscores the potential of multimodality treatment, offering a high‐precision, localized treatment that could be beneficial in specific lesions, increasing, even, overall survival.^4^ In this case, SBRT achieved complete metabolic response in the remaining lesions, suggesting that incorporating radiotherapy into the treatment of oligometastatic disease could enhance outcomes.^4^

A critical area that remains underexplored is the optimal duration of maintenance immunotherapy.^5^ Our patient has been on maintenance therapy with nivolumab and ipilimumab for 3 years without evidence of disease progression. While most clinical trials have set a precedent for a treatment duration of up to 2 years, we lack a well‐defined rationale for this specific timeframe.^5^ This becomes particularly relevant in cases where patients demonstrate high benefit and low toxicity over an extended period. This scenario reflects a critical junction in clinical decision‐making, where the balance between the potential for a sustained response from prolonged immunotherapy and the risks of cumulative side effects and financial implications needs careful consideration. The decision‐making process becomes particularly nuanced, requiring individualized assessment and a patient‐centered approach.

In this case report we illustrate the potential of combining dual ICI with chemotherapy and the addition of SBRT in treating oncogene‐negative, PDL1‐negative NSCLC. It also highlights the need for further research to improve the outcomes of this population.

## AUTHOR CONTRIBUTIONS

**Salvador Gamez Casado**: Conceptualization; methodology; writing‐review. **Javier David Benitez Fuentes**: Methodology; writing‐original draft. **Beatriz Álvarez Rodríguez**: Methodology; writing‐review. **Gema García Ledo**: Conceptualization; writing‐review; supervision. All authors critically reviewed the manuscript and approved the final version.

## CONFLICT OF INTEREST STATEMENT

None declared.

## ETHICS STATEMENT

The authors declare that appropriate written informed consent was obtained for the publication of this manuscript and accompanying images.

## Data Availability

The data that support the findings of this study are available from the corresponding author upon reasonable request.
